# Cough in Humans with Spinal Cord Injury: What We Know and What Is Next

**DOI:** 10.3390/jcm15208135

**Published:** 2026-10-20

**Authors:** Niraj Singh Tharu, Aastha Suthar, Alexander Ovechkin

**Affiliations:** 1 Kentucky Spinal Cord Injury Research Center, University of Louisville, Louisville, KY 40202, USA; niraj.tharu@louisville.edu (N.S.T.); aastha.suthar@louisville.edu (A.S.); 2 Department of Neurological Surgery, University of Louisville, Louisville, KY 40202, USA

**Keywords:** cough, voluntary cough, respiratory motor training, neuromodulation, spinal cord injury

## Abstract

Individuals with spinal cord injury (SCI) experience great difficulty while clearing respiratory secretions, resulting in an ineffective cough. Despite advances in respiratory care, there is a lack of mechanism-based rehabilitative intervention specifically designed to restore effective cough after SCI. This narrative review provides an extensive overview of current knowledge on cough, translational research gaps, advances in interventions, and future perspectives for enhancing coughing ability in individuals with SCI. The literature on cough and related studies in SCI population were reviewed and a narrative review was undertaken to report the necessary information that could assist in developing a respiratory rehabilitation framework for improving impaired cough capacity after SCI. This review highlights the need for introducing cough recovery program into pulmonary rehabilitation. Future research should adopt a coordinated translational framework that integrates improved understanding of voluntary cough mechanisms, standardized assessment of cough effectiveness, careful characterization of neurological and respiratory impairment, and rigorous evaluation of rehabilitative interventions to develop evidence-based strategies for respiratory recovery following SCI.

## 1. Introduction

**Figure 1:**
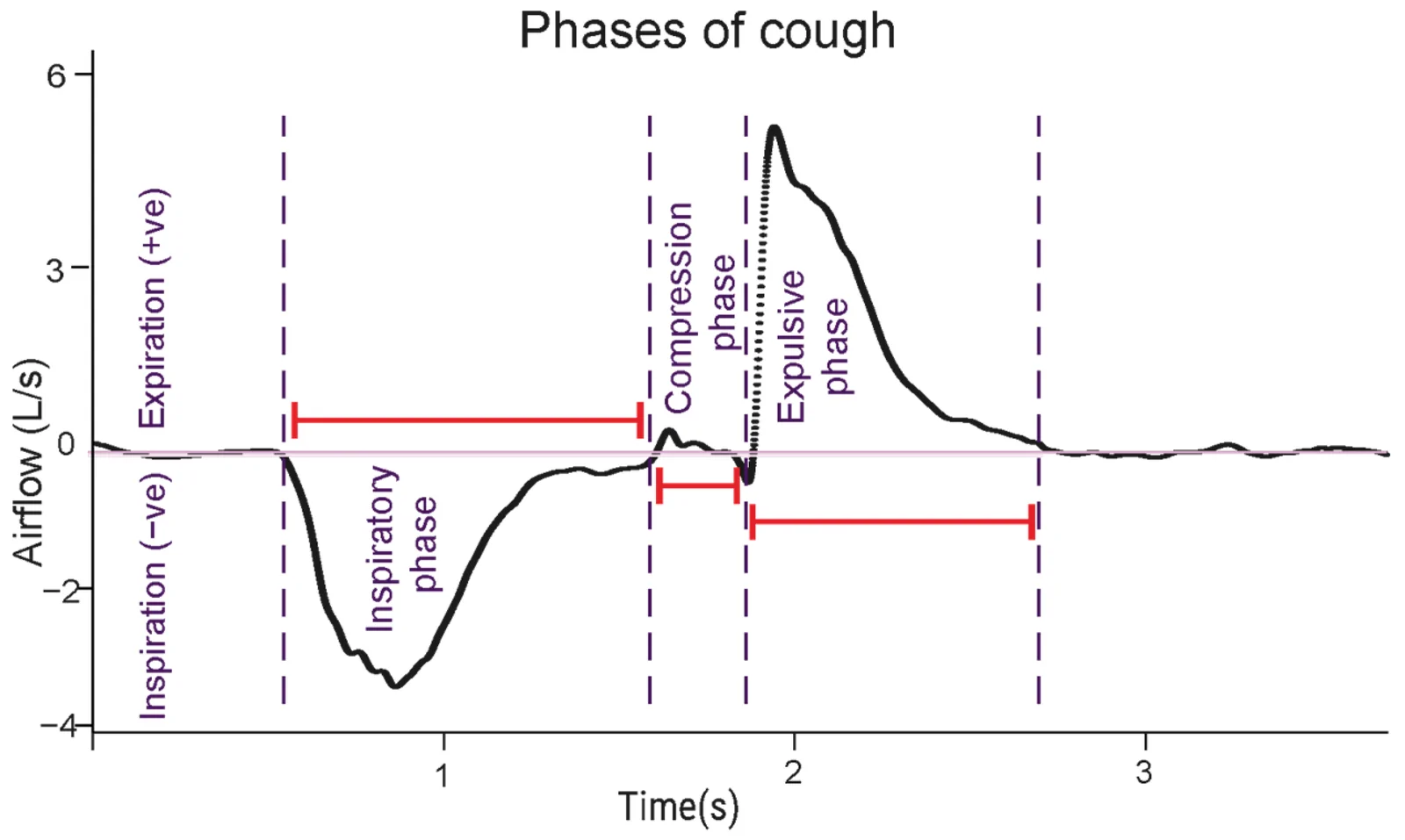
Phases of cough are described as follows: negative airflow is inspiration (inspiratory phase), positive airflow is expiration (expulsive phase), and glottic closure represents the compression phase.

Cough is characterized by the unique sound produced by the quick expulsion of air after forceful expiration against a closed glottis [1]. In addition, coughing is a coordinated protective response that clears the airway of foreign materials by producing quick and robust airflow [2]. Concurrently, in reaction to aspiration, cough is elicited as a defensive response, which generates high-velocity airflows, which in turn induce shear forces that remove and expel the foreign materials from the respiratory tract [3]. Clinicians define coughing as the forceful exhalation of air from the lungs accompanied by a distinctive sound. Coughing, according to physiologists, is a three-stage motor act: the inspiratory phase involves pushing air in through the airways, the compressive phase involves forcing air out through a closed glottis and creating increased intrathoracic pressures, and the expiratory phase involves abruptly opening the glottis and generating rapid airflow during expiration (Figure 1) [4]. Therefore, cough lacks a standardized definition and is explained in various terms by researchers and clinicians.

Spinal cord injury (SCI) frequently results in paresis or paralysis of the respiratory muscles, profoundly impairing voluntary motor control of breathing and coughing [5]. The impairment of inspiratory or expiratory muscles significantly impacts an individual’s capacity to produce the forces necessary for coughing [6]. Affected individuals have reduced coughing ability, leading to an ineffective cough. The inability to cough effectively has serious consequences, since these individuals struggle to expel secretions and are susceptible to inspissated secretions, recurrent aspiration, atelectasis, bronchitis, and pneumonia. Respiratory tract infections are a prevalent cause of mortality in this group [7]. However, respiratory rehabilitation measures remain to be fully explored. Despite advances in respiratory care, there is a lack of mechanism-based rehabilitative intervention specifically designed to restore effective cough after SCI [8]. Additionally, this narrative review provides an extensive overview of current knowledge on cough, translational research gaps, advances in interventions, and future perspectives for improving impaired cough in individuals with SCI.

## 2. Methods

### 2.1. Search Strategy

To ensure broad inclusion, the authors initially sought any publication related to cough and respiratory recovery targeting pulmonary rehabilitation, irrespective of the publication title. The literature was searched across five electronic databases, namely PubMed, Ovid, Embase, Science Direct, and Web of Science, to identify studies published in English. Additionally, manual searches were conducted using Google Scholar to retrieve relevant publications, clinical trials, and systematic reviews. Bibliographies of these sources were further screened to identify additional eligible studies. The primary focus of the search was to explore studies that investigated interventions to improve cough function following SCI, including other neurological conditions in both human and animal models. Given the limited number of eligible studies, no restrictions were placed on the publication year or sample size. The literature search was conducted from inception to 31 May 2026.

The search terms used were as follows: spinal cord injury, cervical cord injury, thoracic cord injury, tetraplegia, quadriplegia, cough, voluntary cough, volitional cough, reflex cough, cough peak flow, respiratory paralysis, respiratory muscle activation, electromyography, cough recovery, respiratory recovery, respiratory rehabilitation, pulmonary rehabilitation, respiratory muscle training, inspiratory training, expiratory training, electrical stimulation, neuromodulation, etc.

### 2.2. Search Inclusion and Exclusion Criteria

This narrative review primarily includes both animal and human studies that specifically prioritized cough impairment, cough outcome assessment and rehabilitation measures in the SCI population. Additionally, it also includes research conducted on different neurological conditions that mostly focused on improving cough and respiratory function following respiratory impairments.

The following inclusion criteria were followed to select studies that primarily focused on: (i) cough and respiratory impairment recovery following SCI, including other neurological conditions, (ii) investigations of the neurophysiology, and mechanisms of cough, (iii) cough recovery and respiratory recovery following respiratory paralysis and impairment, and (iv) interventions for improving cough and respiratory function following SCI, including other neurological conditions. In addition, studies that focused on general acute and chronic cough conditions and their interventions intended to relieve those symptoms were excluded.

## 3. What We Know

### 3.1. Cough Phases

There are three main phases of cough: inspiratory, compression, and expulsive and effective coughing requires all three stages [9]. Robust activity of the inspiratory muscles—including the diaphragm, inspiratory laryngeals, and inspiratory intercostals—fills the lungs with air during the inspiratory phase of coughing. During the compression phase, the muscles of the larynx quickly contract in response to an increased contraction of the thoracic muscles (intercostal and triangularis sterni) and abdominals, which close the airway and build intrathoracic pressure. When the airway opens (i.e., larynx), the expulsive phase starts, generating a rapid flow of air (up to 12 L/s in humans). Strong contraction of the expiratory thoracic and abdominal muscles occurs [10], producing enough expiratory air flow to clear the unwanted materials from the airways [11]. In addition, cough efficacy is negatively impacted by any impairment of the inspiratory and/or expiratory muscles, which in turn raises the possibility of pulmonary complications such as pneumonia [12].

### 3.2. Commonly Studied Cough Types

There are primarily two types of coughs that have been extensively studied in humans with SCI namely reflex cough and voluntary cough, while the urge to cough has been investigated in a few studies [13,14,15]. Voluntary or volitional cough is distinguished by deep inspiration followed by a rapid exhalation of air during expulsion. Reflex or non-volitional cough is a reflex triggered by sensory nerves from the region of the vagal afferents [6]. Voluntary cough is significantly different from reflex cough [13]. The urge to cough is triggered when airway sensory nociceptors detect a noxious stimulus [16]. Reflex cough and the urge to cough are inextricably linked [17]. In addition, voluntary cough serves to prevent aspiration and remove secretions whereas, reflex cough functions to safeguard the respiratory tract from foreign substances [13]. A study that investigated reflex and voluntary cough in tetraplegic subjects reported that individuals with SCI have increased sensitivity to cough inducing stimuli; however, the expiratory peak flow produced by voluntary cough was greater than that of reflex cough, indicating that voluntary cough could serve as the primary airway clearance approach for SCI [18]. Voluntary cough is particularly relevant to pulmonary rehabilitation for SCI, as it can be intentionally initiated and modified through supramedullary control, although its neural control remains insufficiently described [19].

The most common type of stimuli that trigger coughing include locally formed substances like mucus, ingested chemicals such as capsaicin, and aspirated materials, including foreign bodies. The non-volitional cough induced by these stimuli may be reflexive or may be preceded by a sense of the urge to cough, which may or may not result in a motor expression of cough, depending on stimulus strength [20]. Furthermore, a cough event could be single or sequential. A single cough comprises the three phases (inspiratory, compression and expulsive) while a sequential cough has two or more cough epochs that occur sequentially after a single inspiratory effort [21,22]. The first cough epoch exhibits behavior similar to that of a single cough, while the second cough epoch is a scaled-down version of the first cough epoch. Thus, coughing is often measured by calculating either expulsive bouts or epochs [22].

### 3.3. Characteristics of Cough

Cough is a three-phase behavior comprising inspiratory, compression, and expulsive phases. Two factors make the inspiratory phase of a cough essential: first, it helps to differentiate the cough reflex from the expiration reflex. Second, the mechanical forces activated by cough during inspiration greatly affect the force and efficiency of the subsequent expulsive effort [4]. The inspiratory period is about 0.65 s long in humans with no respiratory deficits [23]. As the strength of inspiratory muscles diminishes, individuals forfeit their capacity to take a deep inspiration which also impacts coughing [24]. In humans, during the compression phase, the time it takes for the glottis to close is approximately 0.2 s, which varies from 0.09 to 0.34 s, and from 0.04 to 1.01 s, as reported in the literature [23,25]. It is believed that the efficacy of the compression phase is reflected in the length of the glottal closure, which is characterized by an increase in subglottic pressure before the expulsive phase. As a result, various stages of the respiratory deficits have distinct cough mechanisms, which may explain why the length of glottal closure varies significantly [25]. Furthermore, since the expiratory muscles are isometrically contracted during the compression phase, large intrathoracic pressures are generated. Thus, the muscles are able to keep up the length–tension relationship that is required to produce the increased positive pressures [23].

Expulsive phase begins with the sudden opening of the glottis during which the elevated intrathoracic pressure generated during the compression phase produces strong expiratory airflow [26]. Initially, cough peak flow (CPF) has been commonly measured to assess the cough capacity. It was reported that CPF values at or below 160 L/min are deemed inadequate for clearing airways in individuals with tetraplegia [27], while values lower than 270 L/min are associated with greater secretion accumulation and a higher likelihood of respiratory infection [28]. Values above 360–400 L/min, are considered normal, with the ability to clear airways independently [28]. Although a greater CPF generally indicates an enhanced ability to produce expiratory airflow, it does not necessarily indicate effective airway clearance [29].

Individuals with SCI show significant impairment in cough-related expiratory airflow [30]. Expiratory peak flow (EPF) represents the maximum airflow generated during a forced expiration or cough. In humans with no respiratory deficits, EPF could rise up to 12 L/s [26]. In adults with SCI, EPF has been measured at approximately 3.73 L/s [31], while in children with SCI, it was 2.83 L/s [30]. However, it has been reported that those with EPF value lower than 4.5 L/s are likely to be at higher risk of respiratory tract infection [30]. Additionally, the CPF and EPF values were commonly evaluated in patients with dysphagia, tracheostomy, or those requiring extubation, including those with COPD [29,30]. Therefore, the reported airflow thresholds are clinical reference values whose specific validation in SCI remains uncertain. Specifically, individuals with cervical and upper thoracic injuries may have respiratory muscle paralysis that impairs coughing ability and reduces CPF and EPF, thereby increasing the risk of pulmonary-related infections [31]. The amount of CPF and EPF required to produce an effective cough in SCI is unclear [28]. In addition, CPF and EPF represent only one component of cough, while there are many other factors that need to be considered for an effective cough. Their clinical interpretation also remains limited, warranting future studies to determine whether these values are clinically meaningful.

### 3.4. Neurophysiology of Cough

In the year 1838, Muller discovered from experimental animals that the medulla oblongata excites the muscles which produce contraction of the glottis, and at the same time gives rise to spasmatic actions in the thorax and abdomen. Then the glottis is partly opened, and a loud noise is produced, which he called cough [32]. We still do not know whether the neurons in the brain stem create the cough motor pattern which is responsible for generating normal breathing rhythm or there is a distinct center for coughing. It has been reported that inspiratory and expiratory modulated neurons in the caudal lateral medulla and inspiratory neurons in the region of the nucleus tractus solitarius showed increased firing rates during the different phases of cough. These observations suggested the involvement of respiratory network neurons in cough [33,34].

There are five distinct types of receptors linked to the sensory nerves in the tracheobronchial tree and lungs that have been studied in experimental animals, including humans. These include (i) slowly adapting stretch receptors (SARs), (ii) rapidly adapting stretch receptors (RARs), (iii) pulmonary C-fibers, (iv) bronchial C-fibers, and (v) neuroendocrine bodies. Bronchial constriction activates RARs, while inspiration activates SARs, both of which are intrapulmonary mechanically sensitive afferent nerves. Despite their potential to aid coughing, they are not thought to have a significant role in coughing in humans [35]. C-fibers are subdivided into two categories, with jugular and nodose ganglia being the origins of bronchial and pulmonary C-fibers. Caution should be taken when administering anesthesia, since activation of bronchial C-fibers causes coughing in conscious humans but has no effect on animals that have been anesthetized. This finding raises the possibility that anesthesia suppresses the conscious feeling of airway irritation in animals [35].

Among them, SARs influence the pattern of breathing; however, there is no proof that they have a direct role in coughing, since they were not stimulated by irritant stimuli that elicit coughing in humans. The RARs, also known as irritant receptors, have small diameter myelinated fibers and are the most probable candidates for cough receptors, along with the pulmonary and bronchial C-fiber receptors, which have nonmyelinated afferents. There is proof that RARs are responsible for coughing due to their localization in areas of the airway that are most sensitive to coughing (the larynx and carina), considering the fact that all the mechanical and chemical stimuli that cause coughing also stimulate them, and the fact that numerous epithelial nonmyelinated fibers are linked to myelinated fibers in vagal trunks [32].

It has been proposed that pulmonary stretch receptors (SARs and RARs) play a significant role in the process of coughing by providing sensory information to the cough centers. Evidence for this claim comes from studies conducted on animals in which sulfur dioxide was used to disrupt the stretch receptors. Despite mechanical stimulation of the trachea, these animals did not exhibit coughing behavior. A possible explanation is that RARs in the trachea and proximal bronchi cause coughing, whereas RARs farther down in the lung cause hyperpnea, increased breathing rate, and maybe coughing as well [36].

The possibility that C-fiber receptors contribute to coughing has been the subject of much debate. In fact, when the cat’s pulmonary C-fiber receptors are stimulated, the cough reflex that is caused by mechanical stimulation of the larynx or trachea is inhibited. A more reasonable explanation for cough might be the mediating role of bronchial C-fiber receptors. Their location is ideal, because they share a common response with RARs and pulmonary C-fiber receptors in terms of mediators and irritants. Nevertheless, similar to C-fiber receptors in the lungs, there is evidence that bronchial C-fiber receptors suppress coughing [32].

The majority of our understanding of cough neurophysiology is based on studies conducted on animals [35]. Several of these experiments required animals to be in an altered conscious state, such as an anesthetized state [37]. It has been reported that cough in animals involves a respiratory pattern generator and studies have suggested the possibility of a cough pattern generator. Similarly, in humans, researchers have proposed a similar hypothesis suggesting the presence of a cough center in the brain stem which is yet to be confirmed [34,38]. Therefore, results acquired from anesthetized animals assist in identifying neural pathways and mechanisms but have limitations when extrapolated to cough behavior in humans. Given the vast variations between the species, findings from animals may not be directly applicable to humans. In addition, animal studies primarily looked at reflex cough under anesthesia, which differs from the reflex and voluntary cough in humans that use sensory nociceptors and voluntary cues, respectively to initiate the cough response. In humans, the expiration reflex resembles to reflex cough, but the expiration reflex lacks an inspiratory phase and consists of air expulsion following nociceptor stimulation, whereas reflex cough may include an initial inspiration prior to cough expulsion. In addition, voluntary cough comprises an inspiratory phase before the cough expulsion [19,39]. Importantly, voluntary cough, reflex cough and the expiration reflex represent related but physiologically different airway behaviors. Therefore, the neurophysiology of reflex cough may not be considered equivalent to that of voluntary cough. The neural control of voluntary cough following SCI has rarely been studied. Because voluntary cough can be intentionally produced and modified, it represents a potential target for pulmonary rehabilitation that may be used to improve airway clearance [40]. This warrants further investigation in humans to explore the neural control, mechanisms and neurophysiology of voluntary cough, as understanding this concept could help researchers develop cough-specific recovery strategies in those with cough impairments.

### 3.5. Respiratory Muscles for Cough

One of the primary functions of coughing is to create forceful expiratory airflows, which help to clear the intrathoracic airways of foreign substances or excess mucus. Different respiratory muscles work in complex coordination to regulate the cough. There are three separate stages required to produce high linear airflow velocities during voluntary coughing: the inspiratory phase, which involves the muscles of the chest wall and the larynx, the compression phase, which involves the muscles of the larynx and the abdominals, and the expulsive phase, which involves the muscles of the chest wall and the abdominals [6].

During the inspiratory phase, the muscles of the chest wall and diaphragm contract in coordination with the posterior cricoarytenoid and cricothyroid intrinsic laryngeal muscles to widen the thoracic cavity, which increases the volume of the lungs and permits inspiratory airflow [9,23,41]. The diaphragm, external intercostals, and parasternal portion of the internal intercostal muscles are the primary inspiratory muscles, while the sternocleidomastoid, trapezius, and platysma muscles showed minimal contribution [26]. Furthermore, oblique muscle activity is frequently seen in humans when they approach the end of the inspiratory phase of a cough initiated voluntarily [38]. Immediately after the inspiratory phase ends, the thyroarytenoid and lateral cricoarytenoid muscles rapidly adduct the vocal folds followed by the activation of the expiratory muscles, comprising all anterolateral abdominal muscles and the internal and external obliques which are primarily responsible for producing force. The adduction of the vocal folds is known as the compression phase [9]. Because the expiratory muscles are isometrically contracted during the compression phase, enormous intrathoracic pressures are produced. This permits the muscles to maintain the length–tension connection that is required to produce the large positive pressures [23]. Expiratory muscles such as the rectus abdominis are very lightly activated at this stage [42]. The transversus abdominis, external, and internal oblique muscles, as well as the rectus abdominis, are highly contracted throughout the compression and expulsive phases of coughing [4,38]. Triangularis sterni, also known as the transversus thoracis, is generally inactive during tidal breathing but quickly becomes active in response to both volitional and non-volitional expiratory actions including coughing [38]. The primary expiratory muscles during the expulsive phase were thought to be the abdominal muscles and the intercostal muscles of the lower interspaces [43]. However, coughing uses a coordinated effort of the abdominal muscles, (rectus abdominis, external obliques, internal obliques, and transverse abdominis), though there is controversy over whether the rectus abdominis showed less activation during cough than external obliques [10].

### 3.6. Altered and Compensatory Respiratory Muscle Activation During Cough in SCI

Electromyographic signals within the clavicular region of the pectoralis major muscle (inspiratory muscle) were elevated throughout the compression and expulsive phases in individuals with C6 SCI [42]. It is worth noting that the inspiratory phase showed enhanced activation of the rectus abdominis and oblique muscles (expiratory muscles) in children with SCI, rather than the expiratory phase. In addition, individuals with C5-C7 SCI showed activation of the latissimus dorsi (inspiratory muscle) and teres major muscles (expiratory muscle) during the expulsive and inspiratory phases, respectively. It has also been reported that in tetraplegia, the serratus anterior muscles (inspiratory muscle) were found to be active in the expulsive phase when cough was assessed [10]. Muscle activation at inappropriate times might be a sign of dysfunction in the coordination of the muscles used to cough or a compensatory mechanism [30]. This indicates that respiratory muscles may be recruited and activated differently to compensate for muscles that are weak or paralyzed. Additionally, it is unclear how much the relative strength and intensity of motor activation of these muscles would be disrupted after SCI, which might reduce the efficacy of coughing [10]. The activation of a muscle during a specific cough phase in which it is not typically active in non-injured humans may not be considered evidence of abnormality or dysfunction. The presently available evidence is not sufficient to establish whether these altered activation patterns are beneficial, maladaptive, or simply compensatory [10,44]. Further, electromyographic studies in humans are required to investigate specific respiratory muscles to determine whether their activation contributes to enhanced cough performance. The effect of SCI on respiratory muscle activation during cough depends largely on the neurological level and completeness of the injury and the extent to which descending respiratory motor pathways are preserved [45,46]. Higher cervical SCI may impair diaphragm activation and inspiratory function, resulting in reduced inspiratory capacity and a lower volume available before cough, which may limit cough performance. In lower cervical SCI, diaphragm function is generally better preserved, allowing the generation of the inspiratory volume required before cough; however, weakness or paralysis of the thoracically innervated intercostal and abdominal muscles can greatly impair the compressive and expulsive phases [10,46,47]. Individuals with lower cervical injuries may therefore recruit preserved respiratory and accessory muscles to compensate for impaired expiratory muscle function [10]. With lower or incomplete thoracic injuries, greater preservation of intercostal and abdominal motor pathways can contribute to better forced expiratory function and cough performance [46]. Although the neurological level of injury is associated with respiratory impairment, it may not be considered a predictor for respiratory function as the severity of injury and preserved motor pathways could vary among individuals with SCI. Individuals categorized at a similar neurological level may present with distinct levels of respiratory muscle activation and cough capacity depending on the injury characteristics [45,48]. Future studies should consider these factors when investigating voluntary cough to determine specific voluntary cough techniques based on injury characteristics.

### 3.7. Cough Impairments Following Respiratory Muscle Paralysis in SCI

SCI individuals are associated with a 50% reduction in respiratory function, which leads to the inability to clear the airway through coughing [49]. Indeed, ineffective coughing is the underlying cause of diminished airway clearance ability, which accounts for almost 90% of cases of respiratory deficits. Hence, improving the efficacy of coughing is the primary goal of respiratory care for these individuals [24]. Furthermore, a diminished ability to cough as a result of weak respiratory muscles is a leading cause of illness and death in SCI [24,50]. Insufficient strength of the respiratory muscles limits the lungs from expanding to their full potential as well restricts compression to their minimum residual volume, resulting in reduced elasticity of the chest cavity [24]. People who have sustained SCI, usually have a greater decrease in the ability to exhale than inhale, as a result of their respiratory muscle impairment [51]. Consequently, their coughing ability relies heavily on the functioning of the preserved inspiratory muscles during pre-cough inspiratory phase [52]. Cough strength may also be significantly impacted by activation of these inspiratory muscles, specifically the diaphragm and external intercostals, during the inspiratory phase [43].

In contrast, the diaphragm may become totally or partially paralyzed in cases of severe tetraplegia, compromising breathing and coughing. Individuals with chronic tetraplegia are more likely to have respiratory deficits due to a reduced expiratory airflow, which impacts airway clearance [53]. As a result of paralysis of the chest wall and abdominal muscles, a tetraplegic patient’s efforts to cough or exhale forcefully become significantly weaker compared to non-injured individuals. Insufficiency in the muscles that contract and relax during exhalation reduces the rapid compression that occurs during coughing and affects the linear velocity of airflow across the large intrathoracic airways [54]. The clavicular part of the pectoralis major has been observed to be highly active in tetraplegic individuals, which could actively assist in clearing their airways, even if primary expiratory muscles are paralyzed after cervical SCI [55]. Coughing out violently, nevertheless, may have harmful consequences such as rib fracture, laryngeal and airway injuries, and other unpleasant effects [38]. Therefore, an effective coughing technique could be essential in minimizing such consequences and cough must be a fundamental part of the rehabilitation program in SCI [56].

In people with SCI, respiratory muscle paralysis causes diaphragmatic shifts that alter the diaphragm movement. Simultaneously, the pectoralis major and other accessory muscles, assist in raising negative pressure and lowering the anteroposterior diameter of the upper thoracic cavity. This behavior is considered paradoxical breathing [42]. Paradoxical breathing occurs when the chest moves inward during inhalation and outward during expiration (opposite of normal) in people with SCI because of respiratory muscle impairment [46]. This may lead to discoordination in recruitment of expiratory muscles or serve as a compensatory strategy during cough [57]. Nonetheless, the functional importance of individual compensatory activation patterns remains incompletely recognized. Compensatory activation should therefore not necessarily be understood as impaired coordination without establishing its relationship to cough mechanics.

### 3.8. Outcome Measures for Assessing Cough

Cough severity and its effect on quality of life were first investigated through surveys and questionnaires, such as the Leicester Cough Questionnaire, Visual Analogue Scale for cough [58], Cough Severity Score, Cough Severity Diary, and Cough-specific Quality of Life [59], etc. In addition, they looked at the problems caused by coughing and implemented measures to alleviate such issues. However, these measurement tools were found insufficient to report cough recovery. To evaluate respiratory recovery including cough, studies commonly measured maximal inspiratory pressure and maximal expiratory pressure which quantify inspiratory and expiratory muscle strength, respectively. Spirometric measures were performed including inspiratory capacity, vital capacity, forced vital capacity, forced expiratory volume in 1 s [60,61], total lung capacity, functional residual capacity, residual volume, expiratory reserve volume [62,63,64], tidal volume, maximum inspiratory pressure, maximum expiratory pressure, and maximum voluntary ventilation [6,24,50,65,66]. These measures characterize different aspects of pulmonary function and demonstrate respiratory recovery. Cough recovery was also believed to have occurred following respiratory function improvement when these respiratory evaluation metrics improved. However, these metrics primarily represent pulmonary function and respiratory strength. Therefore, it is matter of debate whether pulmonary assessment measures may not be sufficient to assess cough recovery; specific cough-related biomechanics and physiological metrics should be used to assess cough. Later, researchers examined cough in particular by measuring CPF [53,62,66,67], cough peak expiratory flow rate [25], forced expiratory flow rate [64], peak expiratory flow [65], etc. A more thorough and in-depth examination of cough was undertaken when its three stages—inspiratory, compression, and expulsive phase—were defined. This analysis included inspiratory phase duration, inspiratory phase rise time, inspiratory phase peak flow, compression phase duration, expiratory phase peak flow, expiratory phase rise time, and cough volume acceleration—an indirect measure of cough effectiveness [30,41,68,69]. Cough volume acceleration and expiratory phase rise time are believed to be the appropriate metrics for functional airway clearance, as they may evaluate the shear forces that are essential for airway clearance. Additionally, electromyographic signals of the respiratory muscles were recorded to observe timing and magnitude of muscle activation during cough [30]. The strength of respiratory muscle was thought to be reflected by muscle activation and response. It has been reported that ultrasonographic measurement of diaphragmatic mobility in chronic obstructive pulmonary disease (COPD) patients showed increased diaphragmatic mobility during deep inspiration. This finding demonstrates the potential reliability and responsiveness of diaphragm ultrasonography as a physiological outcome measure, however as it involved COPD, this measure cannot be generalized to SCI [70]. A limited number of articles reported the use of electromyography during coughing in humans [42]. Therefore, the outcome measures that could demonstrate the effectiveness of coughing through specific respiratory muscle strength and cough biomechanics findings warrant further investigation, and future studies which should report the reliability, validity and responsiveness of the cough-related assessment measures. Because there are no definite physiological measures for assessing the effectiveness of coughing, the discussed assessment measures could be combined where feasible for functional and clinically meaningful outcomes. Table 1 provides a summary of various assessment tools used to assess respiratory and cough function, emphasizing key outcome measures, their physical interpretation, and clinical limitations to assist clinicians and researchers in understanding their applicability in clinical settings.

### 3.9. Translational Research Gaps

Recently, one of the important research gaps that should be addressed for translational purposes is the type of cough reflex studied on animal models and humans. Coughing is a voluntary action for humans, and they are also quite sensitive to any irritation in the airways and may suppress it if they want [71]. In addition, animal models rely on external stimuli to elicit the cough reflex; this shows a translational research gap when comparing animal and human cough. The difficulty with animal cough data is that it lacks an essential qualitative component of human cough behavior. Humans experience a sensation or irritation in the throat and/or upper chest that induces an urge to cough, which is under conscious control, and then results in the motor action of coughing [35], while cough assessments in animal models rely on the use of anesthesia and this raises a big debate regarding translational purposes.

Species choice therefore remains important when studying cough mechanisms. It has been shown that mice cannot cough as they cannot generate the energy needed to cough [20]. Therefore, it is possible that researchers are measuring an expiration reflex instead of an actual cough when they use this model [72]. The guinea pig has a particular advantage over other small rodents since it can mimic human coughing physiology and can cough in reaction to stimuli in a conscious state. While rats and rabbits are good laboratory animals for studying chemically induced cough, guinea pigs are more preferred [72]. Similarities have been reported in response to capsaicin and citric acid in both humans and guinea pigs, which validated the guinea pig as a useful model of the human cough reflex [73]. While humans and guinea pigs have many similarities, there are also some key differences. The fact that the guinea pig is an obligate nose breather suggests that its cough reflexes may vary from those of other species such as humans [74]. Studying the cough reflex in anesthetized animals is challenging due to the fact that anesthesia inhibits neuronal conduction and activity in the central nervous system [72]. Additionally, there was no change in respiratory pattern or coughing response to inhalation of capsaicin aerosol. However, these chemical agents elicit a cough reflex in conscious guinea pigs. This suggests that anesthesia should perhaps be avoided when studying the cough reflex in animal models. Moreover, anatomical, sensory, behavioral, and respiratory differences among species suggest that animal models reproduce particular components of human cough rather than complete human cough behavior [75].

There may be characteristic differences between voluntary and spontaneous coughing. A study suggested that coughing can only happen in a conscious state while general anesthesia may reduce the cough response [76]. Research on guinea pigs, or any other kind of experimental animal, needs further investigation before it can be directly translated to humans [26]. A comparison between animal and human coughs, encompassing both voluntary and reflex coughs, requires careful analysis.

Translation to humans with SCI introduces additional complexity. Respiratory impairment after SCI depends on neurological level and completeness of injury and on the preservation of descending respiratory motor pathways. Consequently, two individuals with similar neurological levels may demonstrate different respiratory muscle recruitment and cough performance depending on injury completeness, spared descending connectivity, chronicity, and compensatory recruitment of preserved musculature [46,48]. These SCI-specific characteristics should therefore be incorporated when translating mechanisms identified in experimental models into rehabilitation interventions. Animal and human investigations should also be considered different but complementary stages of translation. Animal models remain necessary for mechanistic questions that cannot readily be addressed in humans. Therapeutic approaches that have demonstrated feasibility and physiological effects in humans should progress toward appropriately designed controlled clinical studies rather than additional cross-species comparison alone [48,75]. Future translational research should use animal studies to resolve fundamental mechanisms of respiratory motor control and coughing, followed by mechanistic human studies and appropriately designed controlled clinical trials to determine the physiological and clinical effectiveness of cough rehabilitation strategies in individuals with SCI.

## 4. What Is Next

### 4.1. Evaluation of Cough

Cough can be initiated for examination in two ways: first, it can be induced to create a reflex cough or urge to cough response. This can be achieved by presenting a chemical stimulus to the subject. Chemical agents, such as acidic solutions and nebulized water, have been used. Secondly, a cough may be assessed by directing an individual to voluntarily cough. The common method for assessing cough is through airflow analysis using a pneumotachograph [9]. In order to compare the cough impairments between normal individuals and those with neurological diseases including SCI, researchers have looked at respiratory and cough-related parameters like CPF, inspiratory phase peak flow, expiratory phase peak flow, inspiratory phase duration, compression phase duration, cough volume acceleration, and maximum expiratory pressure [13,38,77,78]. Electromyography has also been used to record the muscle responses when cough was assessed. Studies have focused on the activation of the inspiratory and expiratory muscles, with findings indicating alterations in the magnitude of activation while coughing [10,42]. While there has been some research on cough-related parameters that may indicate cough effectiveness, there is also a lack of studies using electromyographic measures that have targeted motor recruitment and muscle activation pattern during coughing [30,79]. Reporting these important aspects would provide additional evidence for the development of rehabilitation strategies specifically for improving cough efficacy. Indeed, more detailed evaluation of cough is required to understand the variabilities and address the research gap between animal and human experimental models for clinical translation and the development of treatment approaches.

### 4.2. Develop Mechanism for Voluntary Cough

The mechanism of the voluntary cough is still largely unknown, although there has been a dramatic increase in research in the last 10–20 years in both clinical and scientific interest in coughing [1]. A human being is very sensitive to any discomfort in their airways, has the ability to actively control their cough, and can cough when desired to. This shows that the fundamental cough neural circuit is very responsive to behavioral and cognitive influences. Coughing is a voluntary action, although its neural basis and volitional mechanism are rarely explored [71].

While the mechanisms of reflex cough have been reported in the literature [37,38], the mechanism of voluntary cough has remained unexplored. The voluntary cough could be the primary method for clearing airways in people with impaired cough, such as those with SCI [6]. However, the expiratory airflow produced is insufficient for adequate airway clearance; hence, the cough is not effective. Researchers in this area would benefit greatly from a better understanding of the mechanism underlying voluntary cough if they could get beyond the focus on reflex cough, which has been the primary interest of most studies in both animals and humans [13,20]. People with reduced coughing abilities may benefit from knowing the mechanisms underlying volitional cough in order to develop a therapeutic strategy for improving their reduced cough capacity [41]. When coughing voluntarily, individuals with decreased respiratory muscle strength are unable to generate sufficient intrathoracic pressure to induce dynamic airway compression, and therefore, fail to create a high-pressure gradient in the airways. In the absence of such a gradient, patients will not be able to develop a flow of air at high speed during cough [80]. Thus, development of further evidence in understanding of voluntary cough mechanism might help improve cough efficacy.

### 4.3. Cough Effectiveness in SCI

Neurologically intact humans have the ability to cough and expel unwanted materials and foreign substances from the respiratory airways. Individuals with SCI exhibit paralysis of the respiratory muscles and diminished cough efficacy, resulting in a failure to produce an effective cough [51]. Patients with this problem are at increased risk for aspiration and infections of the respiratory tract, in addition to experiencing physical discomfort that includes difficulties clearing secretions and choking. Indeed, respiratory infections account for a major cause of fatalities in this group of patients [81]. The purpose of coughing is to expel mucus, fluids, and unwanted substances from the respiratory airways by producing high-velocity rapid airflow [3]. The deterioration of pulmonary function due to respiratory muscle weakness might result in an ineffective cough, atelectasis, and other respiratory problems [24].

One key strategy to improve airway clearance is to cough effectively, which requires a high inspiratory volume generated by the inspiratory muscles followed by a higher expiratory flow produced by the expiratory muscles, which is sufficient to remove the undesirable substances from the respiratory airways [31]. An effective cough is essential for maintaining a clear airway and a functional respiratory system [82]. However, cough research has not yet received sufficient attention [25]. It is hoped that a better understanding of cough effectiveness in patients with SCI will help those at greatest risk for respiratory compromise and reduce the incidence and severity of pulmonary complications [56].

### 4.4. Advances in Interventions for Cough Recovery

Various interventions aimed at enhancing respiratory outcomes including cough, comprise mechanical devices, electrical stimulation techniques, respiratory exercises and neuromodulation techniques. Interventions targeting impaired cough in SCI can broadly be distinguished into assistive airway clearance approaches, which enhance cough capacity during their implementation, and rehabilitative or restorative interventions, which aim to produce longer-term improvements in respiratory and cough outcomes. This differentiation is clinically important because an instant rise in expiratory peak flow during an assisted approach may not essentially indicate cough recovery or a lasting therapeutic benefit after the intervention is discontinued.

Initially in acute care, endotracheal suctioning was used for respiratory management to remove mucus in patients with tracheostomy [83]. Later, an external glottic control device was used to facilitate tracheostomy decannulation in patients with neuromuscular disorders, including SCI. This device aided glottic closure, which is often challenging in these patients due to weaker respiratory muscles and reduced glottic function and demonstrated enhanced CPF [84]. However, these devices had limited use among hospitalized patients and needed trained experts for everyday use. Furthermore, mechanical insufflation–exsufflation (cough assist device) was used as an alternative to endotracheal suctioning for managing respiratory secretions in these patients. This device assisted the mobilization of secretions by generating enough positive pressure in the lungs and clearing the airways via the natural cough reflex. Patients preferred this device over endotracheal suctioning because of its efficacy, reduced discomfort, and user-friendliness for long-term use by patients and family caregivers [85]. In addition, this device was assessed to determine its impact when combined with the manually assisted cough approach in people with SCI. This study involved 15 cervical SCI subjects and reported substantial enhancements in airflow outcomes with the combination therapy compared to mechanical insufflation–exsufflation alone; however, no carryover effects were detected across five treatment days. These findings suggest that benefits were primarily observed during application rather than representing restoration of unassisted cough. Despite the small sample size, the study primarily evaluated airflow outcomes rather than airway clearance, therefore its clinical significance is still questionable [86].

Functional electrical stimulation (FES) was introduced in SCI to enhance respiratory function including cough through stimulation of the abdominal muscles which play an important role during both expiration and coughing [87]. FES uses electrical impulses delivered to the abdominal muscles via electrodes, replicating normal muscular contractions and markedly enhancing peak expiratory flow rate by mimicking the natural cough response [88], thereby producing a more robust and efficient cough [87,88]. Research has shown that FES may lessen the risk of respiratory problems while improving respiratory and cough outcomes [61,63]. A randomized crossover trial involving 15 C4-T5 SCI participants showed acute increases in peak expiratory flow and respiratory outcomes, while whether FES-induced physiological improvements persist over an extended period of time remains insufficiently established [63]. Further, a study used Functional Magnetic Stimulation (FMS) to improve the efficacy of coughing in individuals with weak expiratory muscles, including SCI. In order to mimic the normal muscular activity that happens during expiration and coughing, FMS uses a magnetic pulse generator that generates an electric field which excites the neurons associated with the targeted muscles, causing them to contract [89]. FMS demonstrated significant improvements in respiratory-related parameters, particularly in maximal inspiratory pressure and maximum expiratory pressure and increase in cough-related gastric and esophageal pressure. However, the study reported inadequate specific measures of cough airflows; therefore, future research should investigate whether FMS could have clinical benefits for cough efficacy [90].

Karin Postma and colleagues raised attention to the fact that rehabilitation and specific training are necessary to increase the strength of the respiratory muscles. They highlighted the need for specific muscle training for the respiratory recovery in individuals with SCI in order to decrease the possibility of respiratory problems and increase the efficacy of coughing [91]. A study conducted in patients with Parkinson’s disease found that voluntary cough strength significantly changed after four weeks of expiratory muscle strength training (EMST). Specifically, the length of the compression phase and expiratory phase rise time were reduced, resulting in a faster cough. The cough volume acceleration, an indirect indicator of cough efficacy, also increased [69]. Similarly, another study conducted by their team reported that inspiratory muscle strength training (IMST) for 10 weeks dramatically improved inspiratory muscle strength [6]. Both IMST and EMST led to a considerable improvement in maximal inspiratory and expiratory pressures, resulting in positive changes to breathing and cough production, further necessitating the use of both for recovery of respiratory function and cough [66]. In addition, researchers developed a motor exercise paradigm such as respiratory motor training (RMT) which combines both IMST and EMST, to increase the inspiratory and expiratory muscles’ force-generating ability. Here, the choice of respiratory muscles that are targeted using RMT depends on the desired outcome [6,92,93]. Recent evidence continues to support improvements in respiratory outcomes such as MIP, MEP, FVC, FEV_1_, and peak expiratory flow following RMT in participants with SCI [94]. However, improved respiratory muscle strength may not certainly be understood as improved cough effectiveness. A systematic review conducted on non-injured healthy population reported that EMST showed improvement in MEP without any improvement in cough airflow, demonstrating that gains in respiratory muscle strength may not necessarily translate directly into improved cough biomechanics [95]. Therefore, future studies should determine whether RMT-induced physiological adaptations persist after training cessation and whether they improve secretion clearance or reduce respiratory complications.

Neuromodulation techniques have potential as treatments for recovery of lost functions in SCI [96,97,98] and they have shown potential for improving respiratory functions including cough [34,99,100]. Anthony F. DiMarco and colleagues (2024) investigated the impact of spinal cord stimulation (SCS) on cough effectiveness in a patient with SCI. The intervention involved the surgical implantation of a cough stimulator system, which electrically stimulates the spinal cord to activate the expiratory muscles. The findings suggested that SCS could effectively restore expiratory muscle function and improve cough effectiveness, leading to improved airway clearance and reduced respiratory complications in patients with SCI. Nevertheless, improvements in MIP, MEP, CPF, or other physiological variables should be considered evidence of enhanced respiratory motor performance rather than evidence of improved airway clearance until subsequent functional and clinical outcomes are demonstrated [101]. A case study using SCS at the T9 and L1 levels in a tetraplegic subject generated high airway pressures and expiratory airflows, which assisted the subject in coughing independently, considerably reducing dependency on caregiver-assisted cough techniques [77]. In addition, these studies usually involved small, selected groups, and SCS use requires a surgical procedure and have device-related considerations.

Thus, non-invasive neuromodulation, particularly transcutaneous spinal cord stimulation (tSCS) may be used as an alternative and it is increasingly being used in investigations of respiratory motor training [100]. A study using tSCS in individuals with cervical SCI applied stimulation at the C3-4 and T9-10 levels in combination with IMST produced improvements in respiratory outcomes including respiratory muscle strength compared to IMST alone. Although this result offers preliminary clinical support for combining tSCS with respiratory training, it does not establish that tSCS restores effective voluntary cough or improves secretion clearance, because cough-specific measures and airway clearance outcomes were not assessed [100]. Therefore, we believe that a combined approach involving neuromodulation and RMT could offer potential benefits for enhancing cough performance and aiding cough recovery. The rationale for combining these approaches is therefore that neuromodulation may increase the responsiveness of residual spinal respiratory circuitry while, repetitive respiratory training may provide task-specific motor activity capable of promoting activity-dependent plasticity [34]. However, this mechanistic rationale should be distinguished from direct evidence demonstrating synergistic clinical effects on cough recovery. The proposed integration of neuromodulation with RMT should therefore be considered a testable therapeutic hypothesis rather than an established synergistic treatment for cough recovery.

### 4.5. Clinical Safety and Applicability

Clinical implementation of interventions designed to improve cough and respiratory function following SCI requires consideration of safety, patient selection, treatment tolerance, and feasibility in addition to physiological efficacy. Individuals with SCI represent a heterogeneous clinical population, and respiratory impairment varies according to neurological level and completeness of injury, preservation of descending respiratory pathways, time since injury, respiratory muscle involvement, and associated pulmonary impairments [46]. Mechanical insufflation–exsufflation may cause discomfort and pressure related complications in susceptible patients and requires individualized pressure settings and monitoring for adverse effects [29]. The electrical stimulation may cause skin irritation, alter autonomic responses and should be applied cautiously in individuals with implanted electronic devices, impaired sensation, skin vulnerability, or autonomic instability [102]. RMT also requires sufficient medical stability and individualized loading threshold to avoid excessive fatigue or intolerance [103]. Invasive spinal cord stimulation additionally requires surgical implantation and carries procedure- and device-related risks, including the risk of infection and autonomic dysreflexia; therefore, careful risk–benefit assessment and patient selection should be priorities. Although non-invasive neuromodulation avoids implantation, it still requires systematic safety monitoring of skin tolerance, stimulation discomfort, cardiovascular stability, and autonomic responses [77,100]. Therefore, these emerging approaches should be introduced through individualized assessment and monitored rehabilitation rather than assumed to be universally appropriate for individuals with SCI. In addition, emerging interventions, specifically neuromodulation and combined neuromodulation-RMT approaches, should therefore not yet be considered universal components of standard respiratory rehabilitation. Their implementation into standard care will need robust evidence regarding safety, effectiveness, feasibility, durability, and clinically meaningful outcomes across representative SCI populations [46,100]. Therefore, selection of cough and respiratory interventions after SCI should follow an individualized clinical framework rather than a single standardized approach.

### 4.6. Future Perspectives

Despite advances in respiratory rehabilitation and airway-clearance interventions following SCI, several fundamental questions remain regarding how cough recovery should be measured, which interventions provide clinically meaningful benefits, how long these effects persist, and which individuals are most likely to respond. Future research should therefore progress beyond demonstrating short-term changes in isolated physiological variables toward a structured translational framework incorporating standardized assessment, adequately controlled intervention studies, patient-centered outcomes, long-term follow-up, safety monitoring, and individualized treatment selection.

Cough is a coordinated behavior involving inspiratory, compressive, and expulsive phases; therefore, assessment based exclusively on peak airflow values may not fully characterize impaired cough physiology [19]. Future SCI studies should establish standardized protocols specifying participant position, measurement interface, cough instructions, number of repetitions, preceding lung volume, and whether cough is voluntary, reflexive, or assisted. Methodologically rigorous comparative trials are required to determine the effectiveness of emerging rehabilitation strategies [104]. Future studies should use adequately powered multicenter designs and clearly characterize neurological level, injury completeness, chronicity, ventilatory dependence, baseline cough capacity, and relevant cardiopulmonary comorbidities. Automated cough-detection algorithms using acoustic and wearable sensor data have demonstrated the feasibility of identifying cough events objectively, although performance varies according to sensing modality, recording environment, and population [105]. Wearable technologies and artificial intelligence should be investigated for specific cough-related applications rather than as general technological additions to rehabilitation. For SCI, these data could potentially complement episodic expiratory peak airflows, spirometry, respiratory pressure measurements by identifying changes in cough frequency or respiratory patterns and documenting responses to respiratory training.

Respiratory impairment following SCI is heterogeneous even among individuals with apparently similar neurological injuries [46]. Future studies should therefore investigate physiological and neurological predictors of treatment response rather than relying exclusively on group-average effects. Current evidence demonstrates that RMT can improve respiratory outcomes following SCI [103], while preliminary human studies suggest that tSCS can modulate respiratory function and may be combined with respiratory training [100]. Neuromodulation combined with RMT represents a particularly important hypothesis for future investigation because spinal stimulation may facilitate residual respiratory motor circuitry while task-specific respiratory training provides repeated motor activation. Nevertheless, this potential interaction should currently be considered mechanistically plausible and supported by preliminary respiratory evidence rather than an established treatment for cough recovery [100]. Our recently published review reported immediate and post-intervention effects of neuromodulation modalities which enhanced respiratory and cough-related outcomes after the intervention [79]; however, immediate or post-training improvement does not establish that respiratory recovery persists after treatment withdrawal. Future studies should therefore include predefined follow-up assessments after the completion of treatment to determine the retention of improvements in respiratory muscle strength, cough biomechanics, functional airway clearance, and clinical outcomes. Long-term studies of SCS for cough have demonstrated potential clinical benefits but also reinforce the importance of careful patient selection and safety surveillance [7].

## 5. Conclusions

This review highlights the necessity of developing a respiratory rehabilitation framework to enhance the coughing ability of people with SCI and then introducing a cough recovery program into pulmonary rehabilitation. Effective cough is essential for airway clearance; however, in SCI the extent of cough impairment varies according to neurological level, injury completeness, preservation of respiratory motor pathways, and residual respiratory muscle function. Therefore, developing evidence-based cough rehabilitation requires a better understanding of voluntary cough neurophysiology and the mechanisms underlying preserved and impaired respiratory motor control following SCI. Future research should prioritize standardized assessment of cough biomechanics and respiratory muscle activation while distinguishing physiological improvements from clinically meaningful outcomes, including secretion clearance. Although RMT may improve respiratory muscle strength and pulmonary function, and spinal neuromodulation has demonstrated potential to facilitate respiratory motor output and cough-related function, evidence supporting their combined use for cough recovery remains preliminary. Further studies are therefore required to establish efficacy, safety, durability, and patient-specific responsiveness. In addition, advancing cough rehabilitation following SCI will require a coordinated translational framework that integrates an improved understanding of voluntary cough mechanisms, standardized assessment of cough effectiveness, careful characterization of neurological and respiratory impairment, and rigorous evaluation of rehabilitation interventions to develop evidence-based strategies for respiratory recovery following SCI.

## Figures and Tables

**Table 1:** Assessment measures for respiratory and cough function.

Assessment	Outcome Measures	Physiological Interpretation	Clinical Limitation
Respiratory muscle strength	MIP, MEP	Inspiratory and expiratory pressure-generating capacity	Improvement does not indicate effective cough or airway clearance
Pulmonary function	VC, FVC, FEV_1_, TLC, RV, IC	Lung volumes, ventilatory capacity, pulmonary mechanics	Indirect assessment of cough based on injury characteristics and respiratory mechanics
Cough biomechanics	CPF, EPF, cough inspired and expired volume, inspiratory and expiratory phase duration, inspiratory and expiratory phase rise time, cough volume acceleration	Inspiratory, compressive, expulsive phase performance	Indirect measure of cough effectiveness
Neuromuscular activation	sEMG	Timing and magnitude of respiratory muscle activation	Possibility of crosstalk and activation does not necessarily establish mechanical contribution

(MIP: maximal inspiratory pressure; MEP: maximal expiratory pressure; VC: vital capacity; FVC: forced vital capacity; FEV_1_: forced expiratory volume in 1 s; TLC: total lung capacity; RV: residual volume; IC: inspiratory capacity; CPF: cough peak flow; EPF: expiratory peak flow; sEMG: surface electromyography).

## Data Availability

No new data were created or analyzed in this study. Data sharing is not applicable to this article.
